# Supplementary material for: CryoEM reveals how the complement membrane attack complex ruptures lipid bilayers
Source: Nat Commun. 2018 Dec 14;9:5316. doi: 10.1038/s41467-018-07653-5 (PMC6294249; doi:10.1038/s41467-018-07653-5)
Supplement: Supplementary file 1 — Supplementary Information [file 41467_2018_7653_MOESM1_ESM.pdf]

CryoEM reveals how the complement membrane attack complex ruptures lipid bilayers

Menny et al.

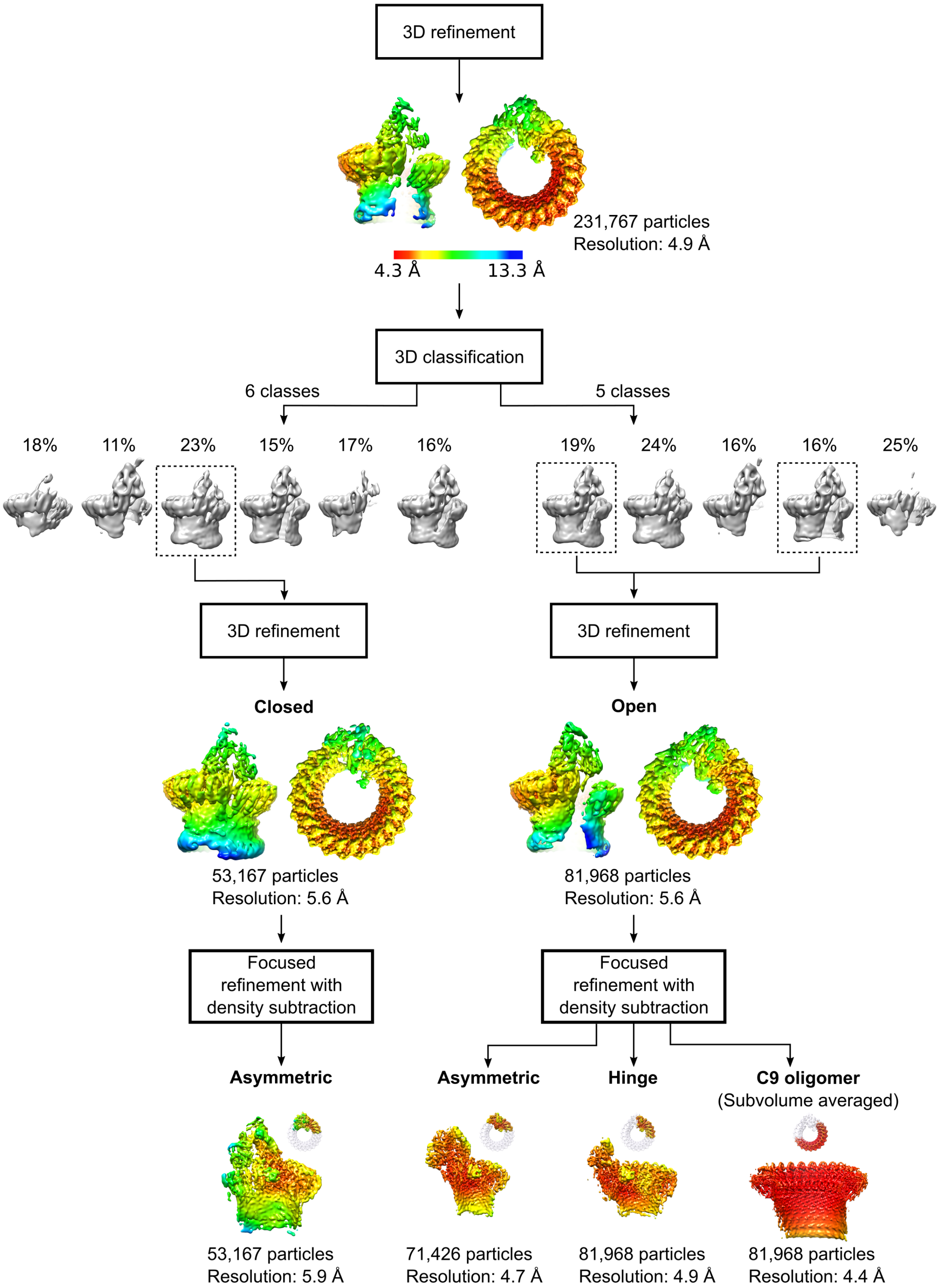

Supplementary Figure 1. CryoEM workflow. Schematic illustrating the image processing workflow. All maps are filtered based on local resolution and visualized using the same color bar scale, ranging from 4.3 Å to 13.3 Å (red to blue). Insets above subvolume reconstructions corresponding to the asymmetric, hinge and C9 oligomer regions show the location of each subvolume within the global reconstruction before density subtraction (transparent surface). The number of particles and average global resolution for each map is indicated.

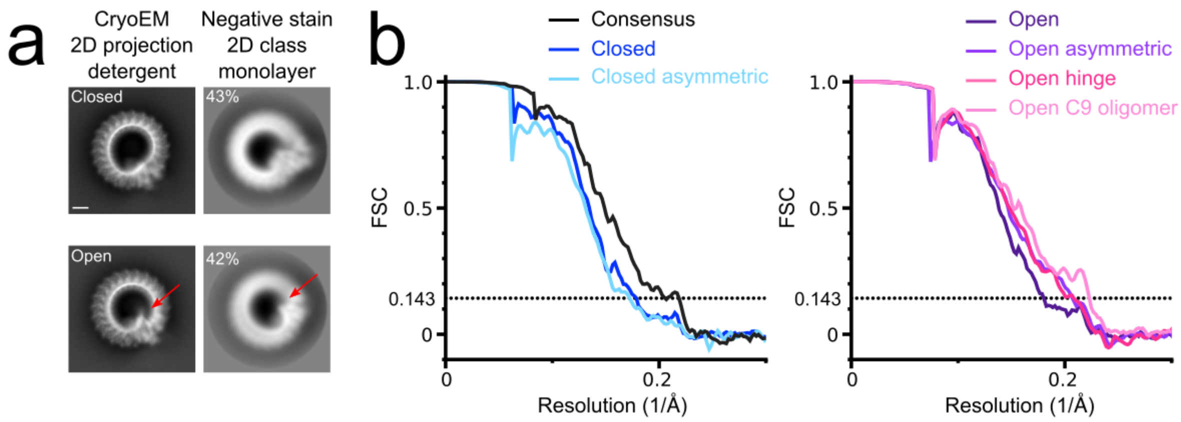

Supplementary Figure 2. Validation of cryoEM. (a) Projections along the axis of the barrel of the open and closed MAC maps agree with 2D class averages of negatively stained MAC pores formed on lipid monolayers. 2D classes are derived from 6889 complete pores. Percentage of particles belonging to each class is indicated. Red arrow indicates density for the "paddle" comprised of C5b6, C7, and C8 that protrudes into the lumen of the barrel in the open conformation. Scale bar, 20 Å. **(b)** Mask-corrected Fourier Shell Correlation (FSC) curves computed from the unfiltered half-maps in RELION.

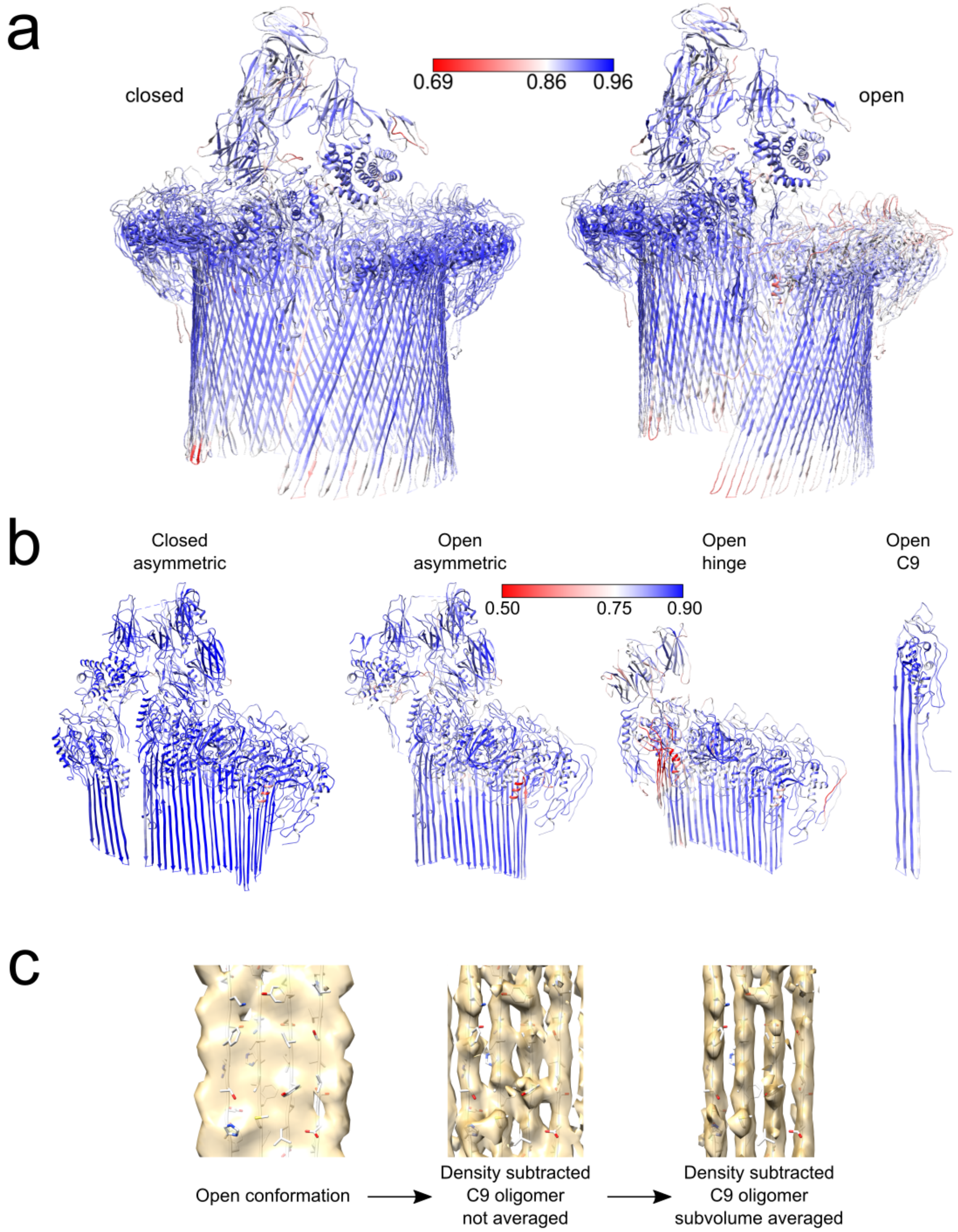

Supplementary Figure 3. Validation of model. (a,b) The local fit of each residue is highlighted using the SMOC score for each protein chain. Segments associated with relatively low scores (in red) reflect areas of higher uncertainty. Scoring was performed for the complete MAC model in the open and closed conformations **(a)**, and for model pieces that were fitted into the higher resolution subvolume reconstructions **(b)**. One C9 monomer was assessed in the subvolume averaged map. **(c)** Overlays of the C9 atomic model with density in the equivalent positions in the open conformation, density subtracted maps, before and after subvolume averaging. All maps are visualized at identical density thresholds.

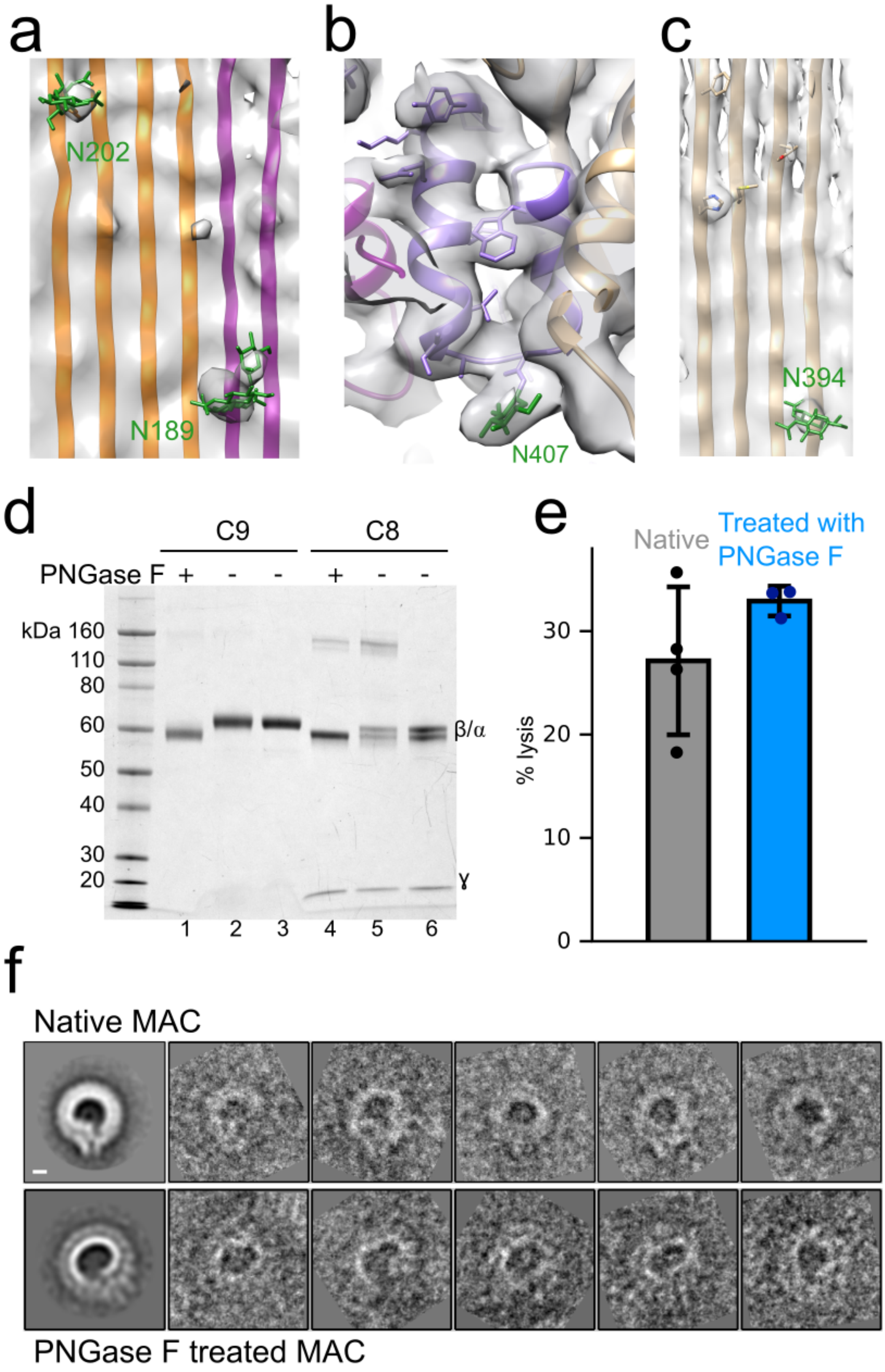

Supplementary Figure 4. Glycans of the MAC. Overlay of the cryoEM map of the open conformation (**a**,**b**: asymmetric subvolume, **c**: C9 oligomer subvolume) with atomic models highlighting density for aromatic side-chains and N-linked glycosylation (sticks, green) of C7 (N202), C8ß (N189), C8α within the CH3 loop (N407) and of C9 (N394). **(d)** Reducing SDS-PAGE gel showing a 48-hour digestion by PNGase F (+ lanes) of N-linked sugars

present on C8 and C9 (lanes 1 and 4). Control C8 and C9 incubated without PNGase F at 37°C for 48 h are shown in lanes 2 and 5, respectively. Fresh untreated C8 and C9 are shown in lanes 3 and 6. **(e)** Percentage lysis of calcein-containing liposomes incubated with either native MAC or MAC assembled from PNGase F-treated C8 and C9. Error bars indicate standard deviation across independent experiments (data points shown as dots). **(f)** Most populated class averages (native, 41% of 706 particles and de-glycosylated, 41% of 711 particles) of negatively stained MAC pores on lipid monolayers (left panel). Right panels show representative single-particles that make up the class average. Pores formed from native complement proteins are shown in the top row; pores assembled from PNGase F-treated C8 and C9 are in the bottom panel. Scale bar, 50 Å.

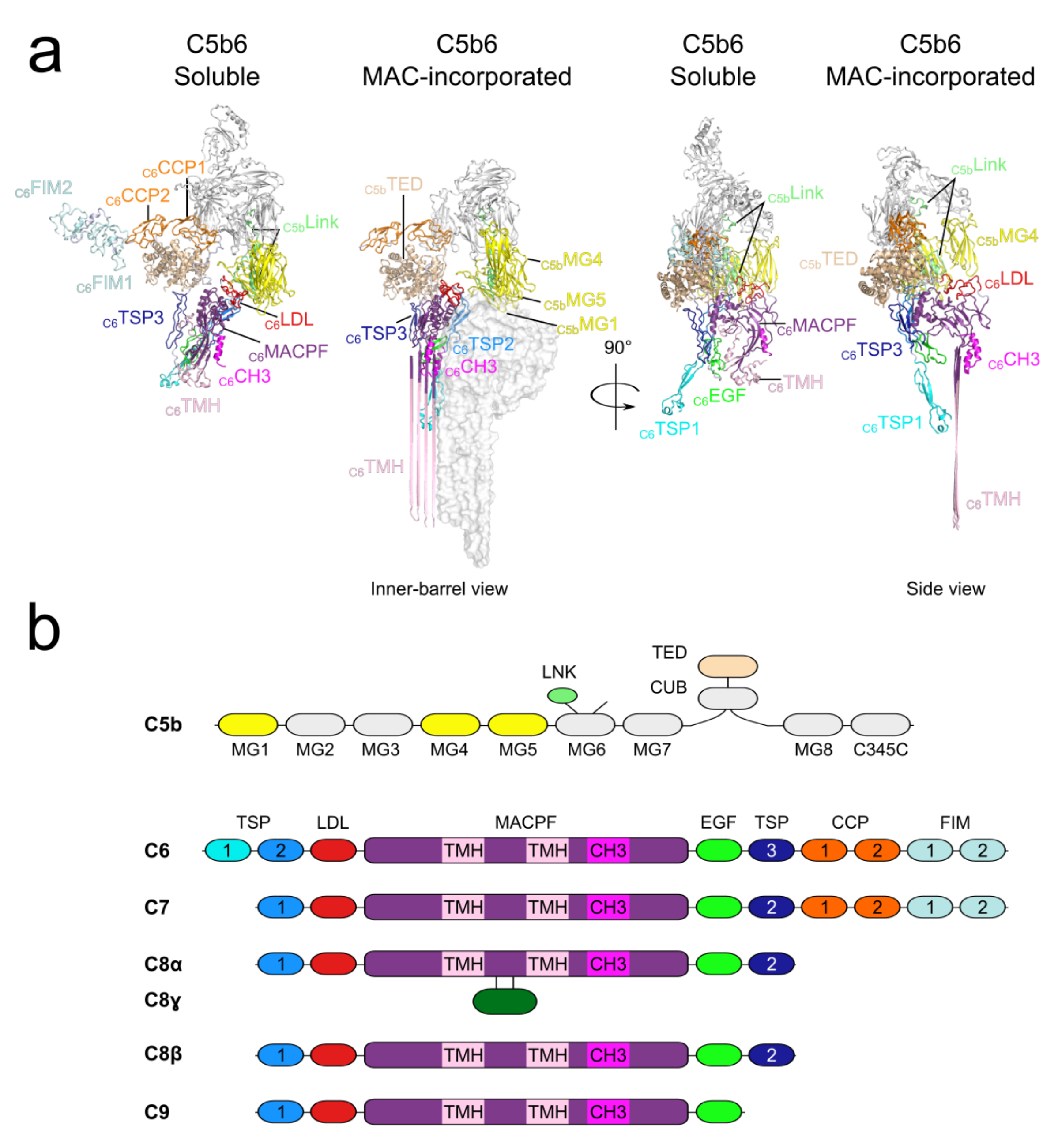

Supplementary Figure 5. Conformational changes of C5b6 upon MAC formation. (a) Two views of C5b6 in its soluble and MAC-incorporated forms. Domains of C5b that interact with C6 (TED, MG1, MG4, MG5, and LNK) are highlighted. All 10 domains of C6 are indicated. CH3 latch (magenta) and TMH regions (light pink) of C6 are shown. Coordinates for C7 and C8β are shown as a semitransparent surface. **(b)** Schematic illustrating the domain organization of complement proteins. Regions of C5b6 highlighted in panel **a** and their structurally homologous counterparts are colored.

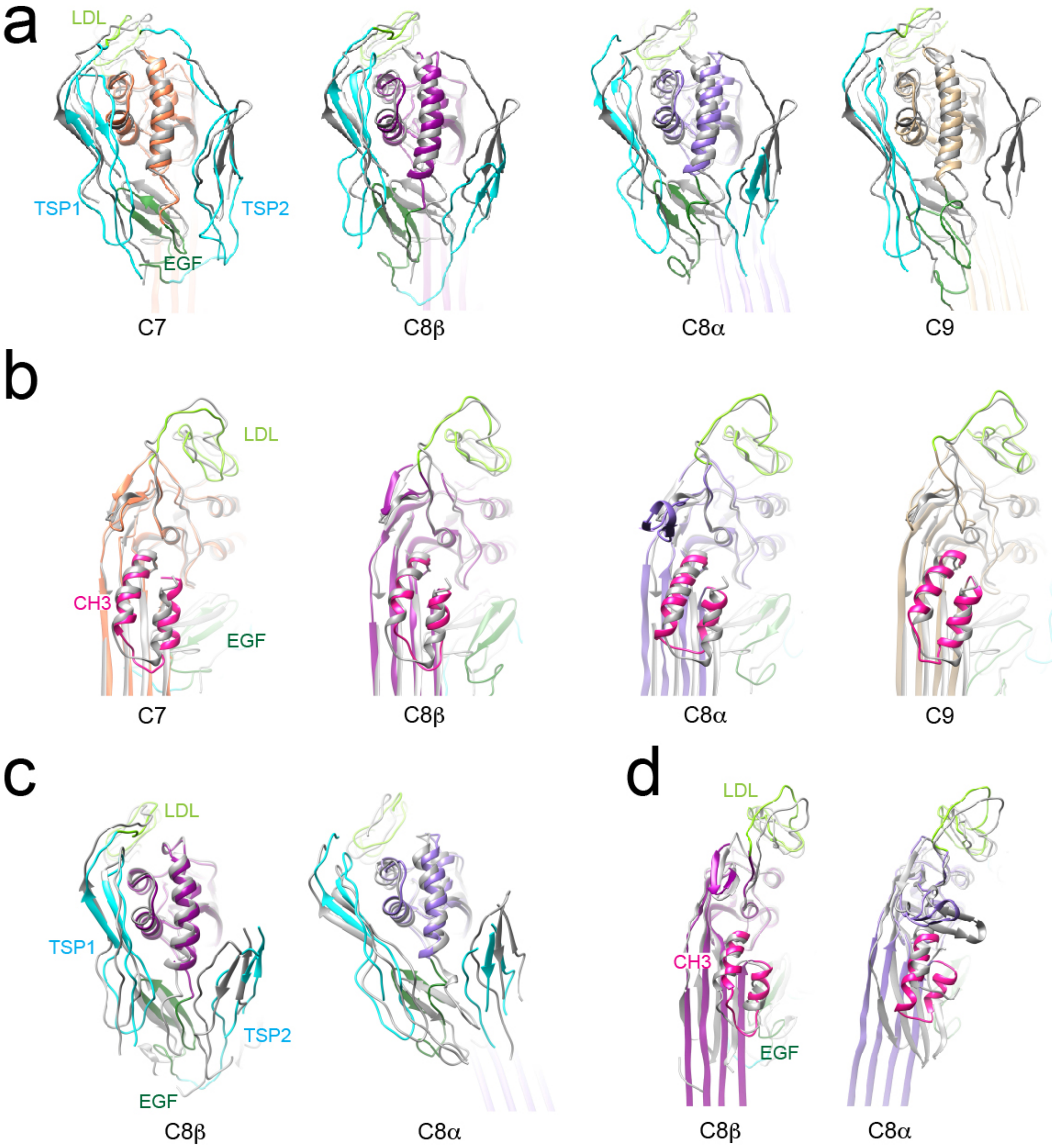

Supplementary Figure 6. Conformational changes of complement proteins. (a) The MAC-associated form of C6 from the open conformation (grey) was superposed on the MAC forms of C7 (orange), C8β (magenta), C8α (purple) and C9 (tan). LDL (light green), TSP (cyan) and EFG (dark green) domains are highlighted. **(b)** Models shown in panel A oriented with a view of the CH3 latch (magenta). **(c)** Superposition of the soluble (grey) and membrane-inserted forms of C8β (magenta) and C8α (purple). LDL, TSP and EGF domains are colored as in panel A. **(d)** Models shown in panel **c** oriented with a view of the CH3 latch (magenta).

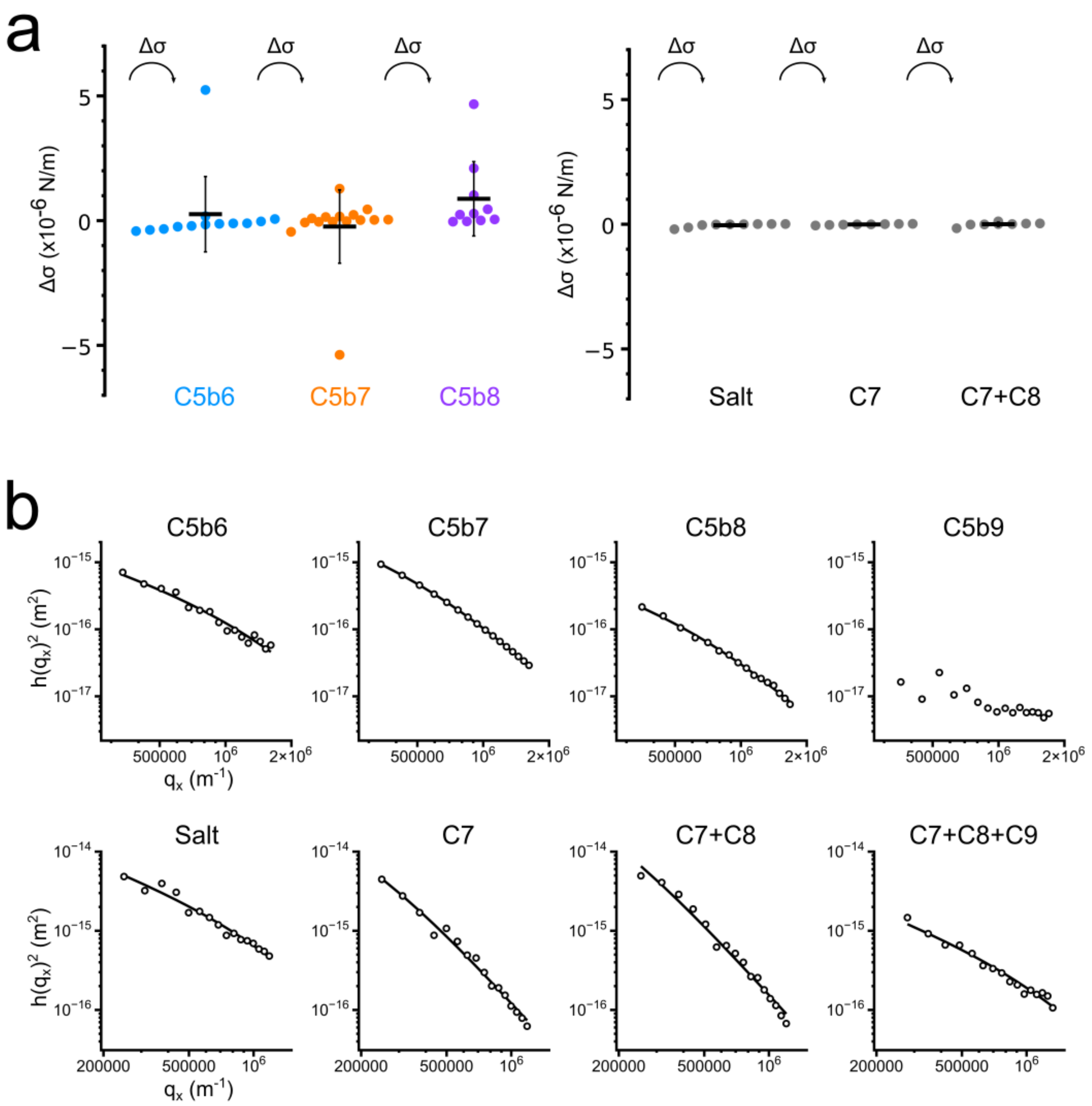

Supplementary Figure 7. Flicker spectroscopy. (a) Variation in GUV tension ($\Delta\sigma$) after each step of MAC assembly (left plot); C5b6 (a complex of C5b and C6), C5b7 (a complex of C5b6 and C7) and C5b8 (a complex of C5b7 and C8). Control conditions where C5b6 is replaced by a salt solution before the sequential addition of C7 and C8 (right plot). Filled circles show individual $\Delta\sigma$ for all GUVs; black lines indicate the mean at each step. Error bars represent standard deviations. **(b)** Representative GUV fluctuation power spectra at each step of MAC assembly or during control conditions. The mean square amplitude ($h(q_x)^2$) of the modes were plotted as a function of mode wavenumber (q_x) (empty circles) and were fitted to the equation (black line), $h(q_x, y = 0)^2 = \frac{1}{L}\frac{k_BT}{2\sigma}\left(\frac{1}{q_x} - \frac{1}{\sqrt{\frac{\sigma}{k_c}+q_x}}\right)$, to extract bending modulus and tension values. The inability to fit the curve after MAC formation was due to reduced membrane fluctuations and a decrease in mode amplitude (see C5b9 plot).

Supplementary Table 1. Cryo-EM data collection, refinement and validation statistics

	#1 consensus (EMDB-0110)	#2 closed (EMDB-0107) (PDB 6H04)	#3 closed (asym) (EMDB-0109) (PDB 6H04)	#4 open (EMDB-0106) (PDB 6H03)	#5 open (asym) (EMDB-0111) (PDB 6H03)	#6 open (hinge) (EMDB-0112) (PDB 6H03)	#7 open ($C9_{avg}$) (EMDB-0113) (PDB 6H03)
**Data collection and processing**							
Magnification	59000x	59000x	59000x	59000x	59000x	59000x	59000x
Voltage (kV)	300	300	300	300	300	300	300
Electron exposure (e–/Å^2)	42-55	42-55	42-55	42-55	42-55	42-55	42-55
Defocus range (μm)	1.75-4	1.75-4	1.75-4	1.75-4	1.75-4	1.75-4	1.75-4
Pixel size (Å)	1.384	1.384	1.384	1.384	1.384	1.384	1.384
Symmetry imposed	C1	C1	C1	C1	C1	C1	C1
Initial particle images (no.)	231,767	231,767	231,767	231,767	231,767	231,767	231,767
Final particle images (no.)	231,767	53,167	53,167	81,968	71,426	81,968	81,968
Map resolution (Å)	4.9	5.6	5.9	5.6	4.7	4.9	4.4
FSC threshold	0.143	0.143	0.143	0.143	0.143	0.143	0.143
Map resolution range (Å)	4.29-13.73	4.54-13.75	4.69-12.29	4.46-13.5	4.37-7.93	4.40-7.60	3.99-6.78
**Refinement**							
Initial model used (PDB code)		2WCY, 3OJY, 4A5W	2WCY, 3OJY, 4A5W	2WCY, 3OJY, 4A5W	2WCY, 3OJY, 4A5W	2WCY, 3OJY, 4A5W	2WCY, 3OJY, 4A5W
Model resolution (Å)		5.6	5.9	5.6	4.7	4.9	4.4
FSC threshold		0.143	0.143	0.143	0.143	0.143	0.143
Model resolution range (Å)		4.54-13.75	4.69-12.29	4.46-13.5	4.37-7.93	4.40-7.60	3.99-6.78
Map sharpening *B* factor (Å^2)	-174	-180	-218	-164	-182	-210	-201
**Model composition**							
Non-hydrogen atoms		97178	97178	102310	102310	102310	102310
Protein residues		12773	12773	12777	12777	12777	12777
Ligands		76	76	80	80	80	80
**Validation**							
MolProbity score		2.06 (100th percentile)	2.06 (100th percentile)	2.35 (99th percentile)	2.35 (99th percentile)	2.35 (99th percentile)	2.35 (99th percentile)
Clashscore		10.81 (97th percentile)	10.81 (97th percentile)	21.25 (91st percentile)	21.25 (91st percentile)	21.25 (91st percentile)	21.25 (91st percentile)
Poor rotamers (%)		1.31%	1.31%	1.25%	1.25%	1.25%	1.25%
**Ramachandran plot**							
Favored (%)		93.6%	93.6%	92.88%	92.88%	92.88%	92.88%
Allowed (%)		99.99%	99.99%	99.96%	99.96%	99.96%	99.96%
Disallowed (%)		0.01%	0.01%	0.04%	0.04%	0.04%	0.04%

Supplementary Table 2. Interface residues of complement proteins

Interface	Charged residues	Polar residues	Hydrophobic residues	Total residues
C6-C7	60	88	67	215
C8α-C9	66	65	82	213
C9-C9	89	66	81	236

Where interface residues are defined as those whose contact distance is equal to or less than 5 Å. Charged amino acids include: ASP, GLU, ARG and LYS. Polar residues refer to: GLN, ASN, HIS, SER, THR, TYR, CYS, TRP. Hydrophobic residues are: ALA, ILE, LEU, MET, PHE, VAL, PRO, GLY.
