# Supplementary material for: CryoEM reveals how the complement membrane attack complex ruptures lipid bilayers
Source: Nat Commun. 2018 Dec 14;9:5316. doi: 10.1038/s41467-018-07653-5 (PMC6294249; doi:10.1038/s41467-018-07653-5)
Supplement: Supplementary file 3 — Description of Additional Supplementary Files [file 41467_2018_7653_MOESM3_ESM.pdf]

Description of Additional Supplementary Files

File Name: Supplementary Movie 1
Description: Morph from open to closed conformations of the MAC as viewed from the membrane. Complement proteins are shown as colored ribbons (C5b, grey; C6, blue; C7, orange; C8β, magenta; C8α, light purple; C8γ, cyan; C9, alternating monomers colored tan and green).

File Name: Supplementary Movie 2
Description: GUV membrane fluctuations. Membrane fluctuations of single GUVs at different time intervals during the step-wise assembly of MAC (top) or control conditions where C5b6 was replaced with buffer (bottom). C5b7 refers to the complex of C5b6 and C7; C5b8 is the complex formed by C5b7 and C8; C5b9 refers to MAC assembly after C9 addition. Scale bar, 10 µm.
